# Characterization of colon cancer cells: a functional approach characterizing CD133 as a potential stem cell marker

**DOI:** 10.1186/1471-2407-12-96

**Published:** 2012-03-20

**Authors:** Meike Schneider, Johannes Huber, Boris Hadaschik, Gabrielle M Siegers, Heinz-Herbert Fiebig, Julia Schüler

**Affiliations:** 1 Department of Urology, University of Heidelberg, Im Neuenheimer Feld 110, D-69120 Heidelberg, Germany; 2 Oncotest GmbH - Institute for Experimental Oncology, Am Flughafen 12-14, D-79108 Freiburg, Germany; 3 Imaging Research Labs, Robarts Research Institute, The University of Western Ontario, London, ON N6A 5K8, Canada

**Keywords:** Stem cells, Colon cancer, CD133, Cancer stem cell like properties

## Abstract

**Background:**

Isolation and characterization of tumourigenic colon cancer initiating cells may help to develop novel diagnostic and therapeutic procedures.

**Methods:**

We characterized a panel of fourteen human colon carcinoma cell lines and their corresponding xenografts for the surface expression of potential stem cell markers CD133, CD24, CD44, CDCP1 and CXCR4. In five cell lines and nine xenografts, mRNA expression of these markers was determined. Tumour growth behaviour of CD133+, CD133- and unsorted SW620 cells was evaluated *in vivo*.

**Results:**

All five putative stem cell markers showed distinct expression patterns in the tumours examined. Two patient-derived cell lines highly expressed CD133 (> 85% of positive cells) and three other cell lines had an expression level of about 50% whereas in long-term culture based models CD133 expression ranged only from 0 to 20%. In 8/14 cell lines, more than 80% of the cells were positive for CD24 and 11/14 were over 70% positive for CD44. 10/14 cell lines expressed CDCP1 on ≥ 83% of cells. CXCR4 expression was determined solely on 94 L and SW480.

Analyses of the corresponding xenografts revealed a significant reduction of cell numbers expressing the investigated surface markers and showed single cell fractions expressing up to three markers simultaneously.

Statistical analysis revealed that the CXCR4 mRNA level correlates negatively with the protein expression of CD133, CD44, CD24 and CDCP1 in cell lines and xenografts.

A lower differentiation grade of donor material correlated with a higher CDCP1 mRNA expression level in the respective tumour model.

*In vivo *growth behaviour studies of SW620 revealed significantly higher take rates and shorter doubling times in the tumour growth of CD133 positive subclones in comparison to the unsorted cell line or CD133 negative subclones.

**Conclusions:**

Our data revealed correlations in the expression of surface markers CD44 and CD24 as well as CD44 and CDCP1 and strongly suggest that CD133 is a stem cell marker within our colon carcinoma panel. Further studies will elucidate its role as a potential therapeutic target.

## Background

There is increasing evidence that the principles of stem cell biology are not only relevant for haematological malignancies but also for solid tumours. This concept includes the hypothesis that tumours consist of heterogeneous populations of cells differing in surface marker expression and growth capacities. Only a small subset of rare tumour stem cells is capable of initiating and propagating tumour formation. These special cells are also thought to initiate tumour metastasis and relapse after therapy [1,2]. A better characterization of tumour initiating cells could lead to improvement of cancer therapies.

In the past few years, subpopulations of cancer initiating cells have been isolated for haematological malignancies [3] as well as for solid tumours such as breast [4], pancreas [5], brain [6], and colon cancer [7,8]. The fundamental problem is to identify and separate tumour initiating cells from more differentiated tumour cells. For this purpose, cell surface antigens are used to characterize different cell populations.

CD133, CD44, CD24, CDCP1 and CXCR4 are five cell surface antigens whose expression is thought to indicate stem cell like properties. CD133 is a five-transmembrane domain antigen with a molecular weight of 120 kDa [9] and is found on stem-like cells of various tissues and cancers like pancreatic, prostate, kidney and colorectal cancer [10].

CD44 is the major hyaluronan receptor and is important for the homing and settling of adult stem cells, metastasizing tumour cells and cancer initiating cells. Upregulated expression of CD44 increases tumour growth and has an anti-apoptotic effect [11].

The expression or lack of CD24 is a hallmark of a wide range of epithelial cancers like pancreatic, prostate or breast cancer [4,11] and has also been used as an indicator for the likelihood of metastasis [12-14]. It may have important roles in migration and invasion by improving interactions between integrins and fibronectin [15].

Multiple SRC-family kinases (SFKs) are activated in carcinoma and appear to have an important role in metastasis and migration of tumour cells. CDCP1 [CUB (complement C1r/C1s, Uegf, Bmp1) Domain-Containing Protein-1] is a transmembrane protein phosphorylated by SFKs, which enables the carcinoma cell to survive. This could be especially beneficial for a tumour stem cell [16,17].

One of the major chemokine receptors expressed by cancer cells is CXCR4, the receptor for CXCL12 [stromal cell derived factor-1 (SDF-1)]. Organs such as the liver or lung produce SDF-1 and thereby increase the risk of developing metastasis by attracting circulating tumour cells [18] expressing CXCR4.

In a panel of colon cancer cell lines growing in 2D culture and as subcutaneous xenografts, we evaluated the expression of these five putative stem cell markers.

## Methods

### Cell culture conditions

The human colon carcinoma cell lines (CCL) HCT116, LOVO, HT29, SW620, DLD1, HCT15, SW480, COLO205, HCC2998, KM12, KM20LZ, and LS174T were obtained from the American Type Culture Collection (Rockville, MD). CCL 269 L and 94 L were established at Oncotest from colon carcinoma patients at the University Hospital/University of Freiburg. All cells were grown in RPMI 1640 medium supplemented with 5% (vol/vol) fetal bovine serum, 1% (vol/vol) penicillin (100 U/mL), streptomycin (100 U/mL), and 1% (vol/vol) L-glutamine (all from GIBCO-BRL, Grand Island, NY). Cells were maintained at 37°C and 5% CO_2_. Media and supplement exchange were performed when 90% confluence was obtained.

### Antibodies

Cell surface marker expression was determined using the following monoclonal antibodies: CD133/1 PE-conjugated mouse anti-human IgG1 (Miltenyi Biotech); CD44 FITC-conjugated mouse anti-human IgG1 (Beckman Coulter); CDCP1 FITC-conjugated mouse anti-human IgG2b (MBL/Mobitec); CD24 PC5-conjugated mouse anti-human IgG1 (Beckman Coulter); and CXCR4 PE-Cy7-conjugated mouse anti-human IgG2a,k (eBioscience). 5 × 10^6 ^cells were incubated with primary antibody or the corresponding isotype control, and the fluorescence intensity was determined by flow cytometry.

### Flow cytometry

The percentage of positive tumour cells was assessed by measurement the fluorescence intensity of the abovementioned cell surface markers. All samples were analyzed on a FC500 flow cytometer (Beckman Coulter) which recorded 100,000-200,000 events per sample. The forward/side-scatter plots and propidium iodide (1 mg/ml, Roche) were used to gate out cell doublets and dead cells, respectively. Cell lines were independently analyzed four times and the mean value ± standard deviation calculated. For the detection of cells expressing more than one antigen, CD133 positive cells were gated on and further analyzed for the expression of CD24 and CD44 or CDCP1 and CXCR4, respectively.

### Mice

For the generation of tumour xenografts, 6-8 week old NMRI nude (NMRI nu/nu) mice were obtained from Charles River, Germany. For tumourigenicity experiments NOD. Cg-Prkdc^scid ^(NOD/SCID) mice were obtained from Taconic, Denmark. [The animals were housed in individually ventilated cages (IVC) set in air-conditioned rooms. The mice had free access to food and acidified water. According to the regulations for animal experiments, individual mice were sacrificed if tumour volume exceeded 1800 mm^3 ^and/or body weight loss exceeded 15%.] All animal experiments were conducted according to the rules of the German Protection of Animals Act (Tierschutzgesetz) and guidelines for the welfare and use of animals in cancer research [19].

### Generation of tumour xenografts

To generate multiple identical xenografts, patient-derived tumours were excised from a donor animal, cut into 4-5 mm diameter pieces and one piece per flank was implanted subcutaneously. Cell-line derived xenografts were induced by subcutaneous injection of 1 × 10^8 ^cells per flank into NMRI nu/nu mice.

### *In vivo *tumourigenecity experiments

The colon cancer cell line SW620 was sorted for expression of CD133 on a MoFlo cell sorter (DakoCytomation). To obtain positive and negative populations, only the top 14% of brightly stained cells or the bottom 21% of dimly stained cells were selected. The unsorted SW620 cell line served as a control. 1 × 10^4 ^or 1 × 10^5 ^cells were injected subcutaneously into five NOD/SCID mice and monitored for their tumourigenecity. The resulting xenografts were analyzed for expression of the antigens by flow cytometry as described above. Tumours were measured either weekly or, for fast growing tumours, twice weekly and volumes were calculated according to the formula a*b^2^/2 where a is the longest diameter and b the perpendicular axis. Group median relative tumour volumes were used for evaluation.

### Preparation of single-cell suspensions

Xenografted tumours were mechanically minced with scissors and scalpels and subsequently incubated with an enzyme cocktail consisting of 41 U/ml collagenase (Sigma), 125 U/ml DNAse (Roche), and 100 U/ml hyaluronidase (Roche) at 37°C for approximately 45 min. The cells were passed through stainless-steel sieves of 200 mm and 50 mm diameter mesh size and then washed. The percentage of viable cells was determined by trypan blue exclusion using a haemocytometer [20].

### Immunohistochemistry

For immunohistochemical processing, sections were dewaxed and endogenous peroxide removed by incubation in 3% H_2_O_2_. For antigen retrieval, the slides were incubated at 600 W in 10 mM Tri-Sodium Citrate (dihydrate) +0,05%Tween in distilled water (pH 6,0) for 20 min. Unspecific binding sites were blocked by applying 5%NGS/1%BSA in PBS for 60 min at room temperature, then a 1:70 dilution of the primary anti-CD133 antibody (Abcam Rabbit polyclonal to CD133 - N-terminal, ab71428) was added for 24 h at 4°C. The Dako-Liquid DAB Substrate Chromogen System was used for further processing and visualization. The sections were counterstained with haematoxylin, dehydrated, and mounted.

### Tumour excision and RNA extraction

For mRNA preparation, tumours were grown in untreated mice until they reached a size of 400 - 800 mm^2^. Following sacrifice, tumours were immediately excised, and tumour pieces free of necrosis were flash frozen in liquid nitrogen. Following mechanical tissue disruption, total tumour RNA was extracted using the RNeasy Mini kit (QIAGEN, Hilden, Germany). Prior to array analysis, one round of T7 promotor-based RNA amplification was performed [21].

### Microarrays, microarray data processing and normalization

Affymetrix^® ^HG-U133 Plus 2.0 mRNA expression arrays were used to determine the expression of 47,400 transcripts, corresponding to 38,500 human genes [22-27]. These arrays have been proven highly reproducible for mRNA expression analysis [23]. CEL result files were pre-processed using the gc-RMA [24] algorithm, after which each transcript was normalized using quantile normalization [25]. Microarray analysis was performed for a distinct colon cancer panel including 9 of the 11 xenografts evaluated for stem cell marker expression and 5 of the above mentioned cell lines. (see GEO nr. GSE35478, http://www.ncbi.nlm.nih.gov/geo/info/linking.html)

### Statistical analysis

Statistical analysis of the co-expressed antigens was performed using the Spearman correlation coefficient (r_S_). The same analysis was applied to the correlation between mRNA and antigen expression. Correlation levels were defined as follows: 0.0 < r_S _< 0.2: no correlation; 0.2 < r_S _< 0.5: weak to medium correlation; 0.5 < r_S _< 0.8: distinct correlation and 0.8 < r_S _< 1.0: strong correlation. For detection of statistically significant differences in the growth behaviour of single tumours, both a *t*-test and a one way analysis of variance was made (ANOVA) Using Sigma Stat Aspire Software (Ashburn).

## Results

### Distinct expression profile of five different surface markers in a colon carcinoma panel

All five surface markers showed differing expression patterns in the colon carcinoma panel. 11 of the 15 examined xenografts were cell-line derived and 4 were patient-derived. For patient data characteristics of the colon carcinoma panel see Table 1. Each cell line or xenograft was evaluated in up to four independent experiments using flow cytometry. Results were highly reproducible, with standard deviations of < 10% within one model for a distinct surface marker.

**Table 1:** Patient data and characteristics of the examined colon carcinoma cell lines and xenografts

cell line	Patient Data					CEA secreting	source
		
		differentiation	gender	age	Dukes Stage	[ng/10^6 cells^]	
HTC116	primary	poorly	male	50	n.k.	1	ATCC
LOVO	metastasis	well	male	56	C	908	ATCC
HT29	primary	middle	female	44	n.k.	yes	NCI
SW620	metastasis	poorly	male	51	C	0,15	NCI
DLD1	primary	middle	male	50	C	0,5	NCI
HCT15	primary	middle	male	50	C	5,4	ATCC
SW480	primary	middle	male	50	B	0,7	NCI
269 L	primary	poorly	male	56	C	n.k.	Oncotest
94 L	primary	middle	male	70	n.k.	n.k.	Oncotest
COLO205	metastasis	poorly	male	70	D	4,1	NCI
HCC2998	primary	well	n.k.	n.k.	n.k.	n.k.	NCI
KM12	primary	poorly	n.k.	n.k.	B	n.k.	NCI
KM20	primary	poorly	n.k.	n.k.	D	n.k.	NCI
LS174T	primary	middle	female	58	B	1994	ATCC
CXF 269	primary	poorly	male	56	C	n.k.	Oncotest
CXF 243	primary	poorly	male	45	n.k.	n.k.	Oncotest
CXF 280	metastasis	poorly	female	56	n.k.	n.k.	Oncotest
CXF 1103	primary	poorly	male	56	n.k.	n.k.	Oncotest

*n.k*. not known

**Figure 1:**
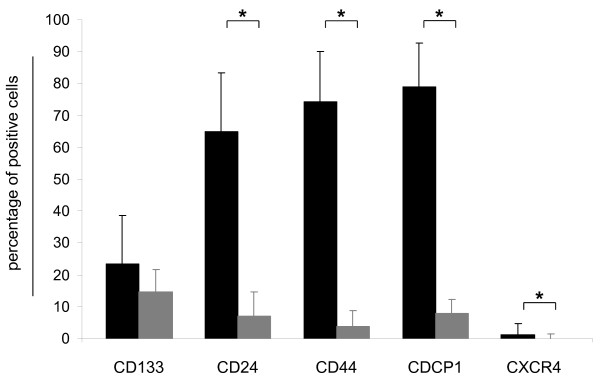
**Antigen expression in the colon carcinoma panel**. Mean expression of CD133, CD24, CD44, CDCP1 and CXCR4 in the colon carcinoma cell line panel (black bars) and the corresponding xenograft panel (grey bars).

**Figure 2:**
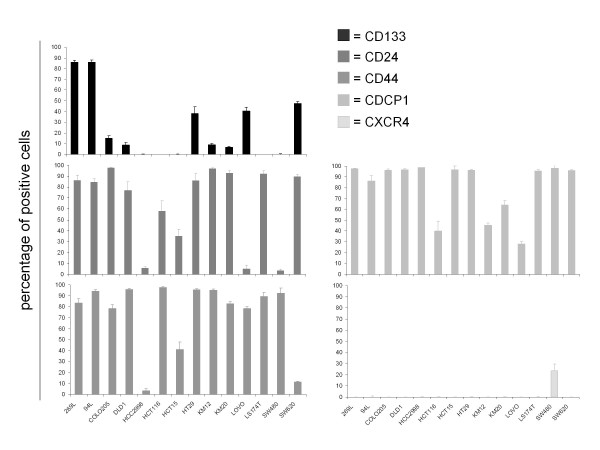
**Expression of 5 different surface markers on a panel of human colon carcinoma cell lines**.

**Figure 3:**
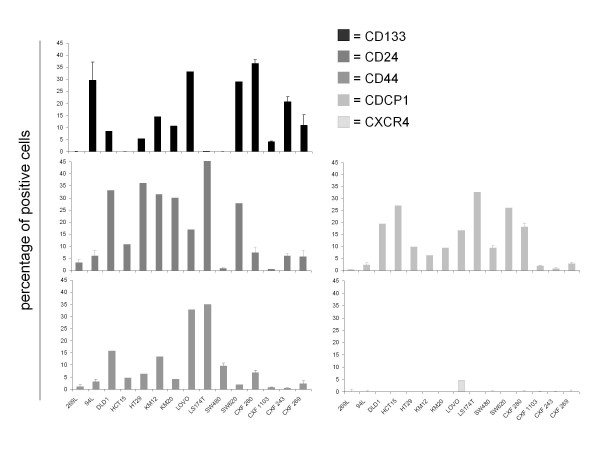
**Expression of 5 different surface markers on a panel of human colon carcinoma xenografts**.

Mean expression of CD133 on cell lines was 23.5 ± 15.2% and 14.5 ± 7.2% on xenografts (Figure 1). With respect to CD133 expression, the cell lines could be subdivided into four groups: five cell lines had a very small subpopulation of CD133 expressing cells (≤ 0.02%); four cell lines expressed 8-15% CD133; in CCL HT29, LOVO and SW620, 50% of the cells expressed CD133; the highest CD133 expression levels were found on Oncotest proprietary cell lines 269 L and 94 L (85.5 and 85.8% respectively) (Figure 2). Examining the colon xenografts, four models revealed very weak CD133 expression ranging between 0 and 0.2%. A group of six tumours expressed CD133 ranging from 4.0 to 14.5%. Another five had expression levels of 20.7 to 36.6% (Figure 3).

Our analyses revealed a mean CD24 expression of 65 ± 18.4% within the cell line and 7.1 ± 7.6% within the xenograft panel (Figure 1). Three groups of cell lines emerged with respect to CD24 expression: 9/14 lines ranged between 76.8% (DLD1) and 97.4% (COLO205). In contrast, three had clearly weaker expression of less than 6%. CCL HCT15 and HCT116 were intermediate, with 35.2% and 58.1% CD24 positive cells (Figure 2). Xenografts CXF SW480 and CXF 1103 displayed CD24 expression ≤ 0.9%. A group of 7 tumours expressed CD24 in a range of 3.3 to 16.9%. A third group within the xenograft panel was characterized by CD24 expression > 24% with a maximum of 53.4% (Figure 3).

CD44 could be detected on all colon cancer cell lines investigated with a mean expression of 74.22 ± 15.73%. Xenografts revealed a mean expression of 3.9 ± 4.9% (Figure 1). The majority of the colon cancer cell lines (11/14) expressed CD44 on almost every cell (ranging 78.3% to 97.6%). Exceptions were HCC2998, which displayed the lowest expression level (3.6%) and SW620 and HCT15 with 11.2% and 41.2% of CD44 positive cells, respectively (Figure 2). Weaker CD44 expression was found within the xenograft panel; the highest expression was 32.8% and 35% on CXF LOVO and CXF LS174T, respectively. 5/15 models ranged between 6.3% and 15.8%, whereas the largest subgroup (eight tumour models) exhibited < 5% CD44 expression (Figure 3).

CDCP1 was the most highly expressed marker detected on our cell line panel comprising 79.1 ± 13.6% of positive cells. Xenografts expressed CDCP1 to 7.9 ± 4.4% on the cell surface (Figure 1). Ten cell lines expressed between 86% and 98.5% CDCP1. On four cell lines, this surface marker was expressed on a lower percentage of cells (< 64%) (Figure 2). With respect to CDCP1 expression, the investigated xenografts could be subdivided into three groups: five models with 0.4% to 2.8% of positive cells; a group of four with expression levels between 6.3% and 9.9%; and the six remaining tumours comprised 16.7% - 32.7% CDCP1 positive cells (Figure 3).

CXCR4 showed very weak expression with a mean value of 1.14 ± 3.6% and 0 ± 1.3%, respectively (Figure 1). The expression ranged between 0 and 0.2% despite CCL SW480 of which 23.5% were CXCR4 positive within the cell line panel (Figure 2). CXF LOVO was the only xenograft for which more than 1% CXCR4 positive cells could be identified (Figure 3).

Statistical analyses revealed no correlations among the investigated surface markers within the cell line panel. However, within the xenograft panel CD24 and CD44 expression correlated distinctly (spearman correlation, r_s _= 0.59, p < 0.001) as did CD44 and CDCP1 protein expression (spearman correlation, r_s _= 0.673, p < 0.001).

Surface marker expression was downregulated in the majority of corresponding xenografts. For CD24, CD44, CDCP1 and CXCR4, the difference in expression level was statistically significant (*t*-test, p < 0.001, p < 0.019; Figure 1). Despite these significant reduction, CD133 expression was up-regulated when KM20 and KM12 tumour cells grew subcutaneously in nude mice. (KM20: 6.6-10.6%; KM12: 9.1-14.9%).

### CD133 positive subpopulation of the xenograft panel included small fractions of double and triple positive cells

**Figure 4:**
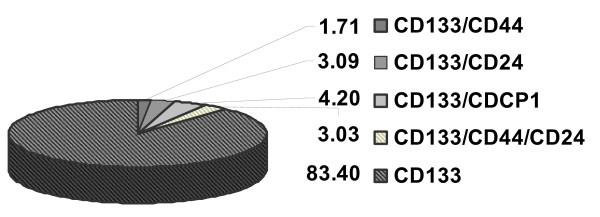
**Mean percentage of double- and triple-positive cells within the CD133+ subpopulation of xenografts**.

The CD133 positive subpopulations within the xenograft panel were further analysed for parallel expression of CD44 and CD24 or CDCP1. Most cells (83.4%) expressed CD133 exclusively on their cell surface. A mean percentage of 3.03 was triple positive (CD133/CD24/CD44). 1.71% (CD133/CD44) and 3.09% (CD133/CD24) were double positive, respectively. 4.2% expressed CD133 and CDCP1 simultaneously (Figure 4).

### CXCR4 mRNA level correlates negatively with protein expression of the other four surface markers

Additional statistical analyses were performed to study the correlation of protein expression (measured by flow cytometry), mRNA expression (measured by gene expression profiling) and patient data within the colon carcinoma panel.

On the mRNA level, CXCR4 expression correlated negatively with protein levels of CD133 and CD24 in cell lines as well as CDCP1 in cell lines and xenografts (Table 2).

**Table 2:** Correlations between different antigens and clinical data

	CCL CD133 protein	CCL CD24 protein	CCL CD44 protein	CCL CDCP1 protein	CXF CDCP1 protein	Patient tumour differentiation	Patient age	
CCL CD24 gene expression							-0,671	spearman correl. Coefficient
							0,00575	p-value
CCL CDCP1 gene expression						0,722		spearman correl. Coefficient
						0,00707		p-value
CLL CXCR4 gene expression	-0,671	-0,615	0,692	-0,622	-0,627			spearman correl. Coefficient
	0,0154	0,0308	0,0113	0,0285	0,0263			p-value

Correlation of protein expression (measured by flow-cytometry), mRNA expression (measured by gene expression profiling) and patient data within the colon carcinoma panel

Another statistically significant correlation could be found between tumour differentiation in the donor patient and the mRNA expression of CDCP1 (spearman correlation, r_s _= 0.722, p = 0.007) in the respective cell line: lower differentiation grade of donor material correlated with higher CDCP1 expression at the mRNA level (Table 2).

### CD133-positive and negative SW620 cells exhibit differing growth capacities *in vivo*

*In vivo *tumourigeneicity experiments revealed that CD133 expressing SW620 cells were more tumourigenic than cells that did not express this cell surface marker. CD133 positive subclones showed significantly higher take rates, as well as shorter doubling and induction times than the unsorted cell line or CD133 negative cells, respectively. At least 1 × 10^5 ^tumour cells were needed for tumour formation as injection of 1 × 10^4 ^SW620 cells induced no xenograft formation within 89 observation days, independent of CD133 surface expression. 1 × 10^5 ^CD133 positive cells induced tumours in all five injected mice within 39 days of tumour cell inoculation. In contrast, in groups receiving either unsorted or CD133 negative cells, tumours occurred later (42 days post-injection) and only in 60% of mice.

**Figure 5:**
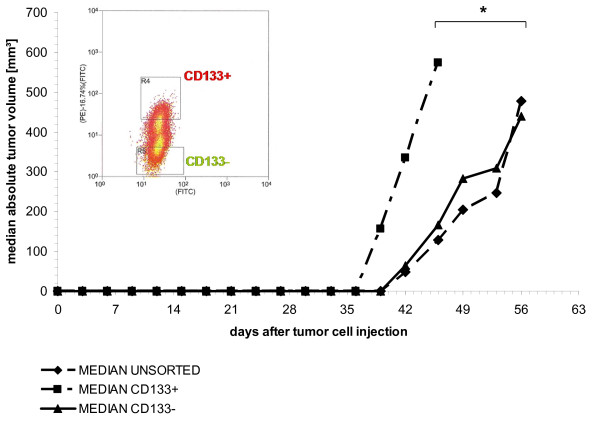
***In vivo *growth behaviour study of SW620 cells**. Growth behaviour of 1 × 10^5 ^CD133+, CD133- and unsorted SW620 colon carcinoma cells injected subcutaneously in NOD/SCID mice.

Calliper measurements revealed a median tumour volume of 572 mm^3 ^46 days after tumour cell injection in the CD133 positive group. This was significantly higher than median tumour volumes of xenografts derived from CD133 negative or unsorted SW620 cells (98.7 and 93.2 mm^3^, respectively, p < 0.001). Thus, the higher tumourigenic potential of CD133 expressing cells was confirmed by these data. No difference in growth behaviour could be determined comparing CD133 negative subclones and the unsorted cell line (Figure 5).

**Figure 6:**
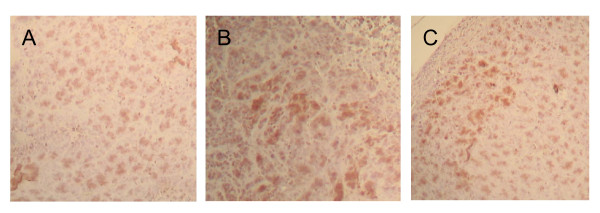
**Immunohistochemistry (IHC) of different SW620 cell fractions**. CD133 expression in xenografts that grew out of unsorted, CD133+ and CD133- subclones of SW620 colon carcinoma cells. A: IHC *α*-hu CD133: SW620 unsorted cells injected subcutaneously (sc) in NOD/SCID mice. B: IHC *α*-hu CD133: SW620 CD133+ cells injected sc in NOD/SCID mice. C: IHC *α*-hu CD133: SW620 CD133- cells injected sc in NOD/SCID mice.

Xenografts from this tumourigenicity experiment were analyzed for expression of CD133 by immunohistochemical staining. No significant difference in the expression profile of this antigen could be shown for different SW620 cell populations (derived from initially CD133+/-/unsorted cells, Figure 6).

## Discussion

A basic problem in cancer research is identifying the cells responsible for tumour formation. Within the cancer stem cell model, there is a small subset of cells capable of initiating and sustaining growth of a neoplastic clone. Tumour stem cells are probably long-lived cells that accumulate cancer-inducing mutations. Furthermore, they have the unique ability to self-renew and, through differentiation, to generate mature non-tumourigenic cancer cells of all lineages. These mature cells appear to constitute the bulk of cancer cells within a tumour.

If the cancer stem cell hypothesis is correct, we have to reconsider treatment regimens that eradicate the bulk of cancer cells, but may not target the cell of origin. These cells are thought to be refractory to classical chemotherapy and responsible for metastasis and relapse. Further characterization of the stem cell population is required to identify potential targets for prospective therapies [26,27].

With this study, we are the first to characterize a large panel of colon carcinoma cell lines and their corresponding xenografts for simultaneous expression of several stem cell markers and thereby identify different cell fractions.

The cell surface antigens examined showed a distinct expression pattern within both colon carcinoma cell line and xenograft panels such that different cell fractions could be distinguished. In general, the cell lines expressed CD133, CD44, CD24, CDCP1 and CXCR4 at higher levels than the xenografts. The cancer stem cell hypothesis suggests that the heterogeneity in a tumour results from ongoing differentiation and the majority of tumour cells lose their proliferative potential during this process of maturation [1,28]. This could be a reason why the antigens are expressed at a lower level *in vivo*. During xenograft development, tumour cells lose their stem cell characteristics and also distinct cell surface antigens. It is already well known for haematological malignancies like multiple myeloma, acute myeloid leukaemia or acute lymphocytic leukaemia that differentiation is driven by cell surface antigens [29]. Aside from differentiation, lower expression of these surface markers might be explained by the influence of the tumour microenvironment. Tumour cells growing subcutaneously in nude mice are exposed to a completely different environment than tumour cells *in vitro*. It is possible that expression of these antigens would have been different if transplanted orthotopically, as it is unclear whether xenotransplantation accurately reflects human stem cell biology or whether the transplanted cells are human cancer cells that have adapted to the mouse environment [30]. The appropriate microenvironment is essential for maintenance of "stemness" as has been shown for hematopoietic stem cells [31], brain tumour stem cells [32] and, more recently, colon cancer stem cells. Vermeulen et al. showed that factors secreted by myofibroblasts also restore the cancer stem cell phenotype in more differentiated colon carcinoma cells. Thus, it appears that "stemness" of colon carcinoma cells is a dynamic quality that can be influenced by the microenvironment [33]. On the other hand, one could also argue that *in vitro *culture conditions select for the maintenance of stem cell properties. In our experiments, we could show that colon cancer cells show very different phenotypes in *vitro *versus *in vivo*, thus suggesting a close relationship between the microenvironment and the existence of different cell types. This knowledge is also crucial for testing new drugs under *in vitro *versus *in vivo *conditions. [Of note, the procedure for preparing single cell suspensions from the xenografts had no statistically relevant influence on antigen expression (see Additional file 1: Figure S7).]

It is not yet known to what extent single antigens are responsible for stem cell maintenance, thus we do not know the functional relevance of these cell surface markers. CD133-expressing colon cancer cells produce interleukin 4 (IL-4) as an autocrine growth factor. IL-4 promotes the induction of anti-apoptotic genes, thus promoting cell survival. Administration of a neutralizing IL-4 antibody improved the efficacy of conventional chemotherapy [34]. These data suggest that elimination of CD133-expressing cells could prove beneficial in the treatment of colorectal carcinoma. Others have described the more tumourigenic capacity of CD133-expressing cells compared to cells that do not express this antigen [7,8]. Also, different Wnt factors affect proliferation and differentiation in CD133-expressing cells [35]. The Wnt signalling cascade has emerged as an important regulator of normal and malignant stem cells in intestinal, hematopoietic and epidermal systems [36]. Since CD133 has thus been characterized as a putative stem cell marker, we aimed to further clarify its exact role via additional functional analysis.

Our data show that CD133 is not expressed by every colorectal tumour cell. In our experiments, tumours that grew out of a CD133-expressing cell population showed enhanced growth behaviour compared to the control group. We could not, however, clearly identify the role of CD133 in this process. Maybe this marker is crucial for the development of the tumour. Alternatively, perhaps it is a marker that does not initiate but rather enhances tumour growth, for example, by means of better tumour vascularization. CD133-expressing progenitor cells in the kidney contributed to better tumour vascularization by differentiating into endothelial cells [37]. Beier et al. demonstrated that glioblastoma cancer stem cells can be either CD133+ or CD133-, suggesting that this marker is not limiting for "stemness" [38]. This fits with our results indicating that tumours also grew out of CD133-negative cells. CD133 knock down experiments would be useful to further explore the functional relevance of this cell surface marker.

CXCR4 is said to play a key role in tumour progression and metastasis in colon cancer [39]. In our study, the mean CXCR4 expression within the xenograft panel was 0.85% with notable expression of > 1% in one of fifteen models. Expression may have been upregulated had cells been injected in a more appropriate microenvironment, e.g., via orthotopic implantation [40,41].

Our study is limited in that the relationship between cell surface antigens and cell properties, like growth behaviour, remains to be further elucidated. In haematological malignancies, identification of cells with different growth capacities is based on cell surface antigens and these exhibit functional heterogeneity [42]. Flow cytometry is a widespread and reliable method to detect antigens on single cells [43]. We analyzed cell surface antigens on single cell suspensions derived from xenografted tumours from our colon carcinoma cell line panel. Our studies revealed that some xenograft cells express more than one of the five surface markers we studied and significant correlations between different markers emerged. CDCP1 is highly expressed in lung and colon cancers, where it is phosphorylated by Src family kinases and involved in anchorage independence of cancer cells [16,44]. These properties are important for tumour progression and metastasis. The correlation we observed between CDCP1 expression and disease stage of donor patients is in line with the results of Uekita et al. who described a correlation between expression and phosphorylation levels of CDCP1 with the invasive potential of scirrhous gastric cancers [45]. These data point to the relevance of this marker as a potential therapeutic target for modulating cancer metastasis.

In addition to correlations between markers and clinical parameters, we found small subsets of double- and triple-positive cells in our colon cancer panel which could comprise a stem cell population and should be analyzed in more detail.

## Conclusions

Our study characterized a large panel of colon carcinoma cell lines and their corresponding xenografts, showing significantly reduced expression of the cell surface markers CD133, CD44, CD24, CDCP1 and CXCR4 *in vivo*. A small subset of CD133-positive colon carcinoma cells additionally expressed CD24, CD44 or CDCP1, so that a correlation in antigen expression can be assumed. *In vivo *growth kinetics provide strong evidence that CD133 plays an important role [within the mentioned surface marker profile]. Further studies will show the functional relevance of these markers in colon carcinomas and assess their potential as therapeutic targets in oncology.

## Competing interests

The authors declare that they have no competing interests.

## Authors' contributions

MS performed most of the experimental work and wrote the first draft of the manuscript and all authors have contributed to, read and approved the final version of the manuscript.

## Ethics committee approval

We performed all actions according to the "Declaration of Helsinki" in its latest version and respected usual data protection requirements.

## Pre-publication history

The pre-publication history for this paper can be accessed here:

http://www.biomedcentral.com/1471-2407/12/96/prepub

## Supplementary Material

Additional file 1 **Figure S7 Expression of five different surface markers on untreated (solid bars) and enzymatically pretreated (shaded bars) colon cancer cell lines 269 L and 94 L**. Click here for file

## References

[B1] Dalerba P Cho RW Clarke MF Cancer stem cells: models and concepts Annu Rev Med 2007 58 267 284 10.1146/annurev.med.58.062105.204854 17002552

[B2] Steeg PS Tumor metastasis: mechanistic insights and clinical challenges Nat Med 2006 12 8 895 904 10.1038/nm1469 16892035

[B3] Wang JC Dick JE Cancer stem cells: lessons from leukemia Trends Cell Biol 2005 15 9 494 501 10.1016/j.tcb.2005.07.004 16084092

[B4] Al-Hajj M Wicha MS Benito-Hernandez A Morrison SJ Clarke MF Prospective identification of tumorigenic breast cancer cells Proc Natl Acad Sci USA 2003 100 7 3983 3988 10.1073/pnas.0530291100 12629218 PMC153034

[B5] Li C Heidt DG Dalerba P Burant CF Zhang L Adsay V Wicha M Clarke MF Simeone DM Identification of pancreatic cancer stem cells Cancer Res 2007 67 3 1030 1037 10.1158/0008-5472.CAN-06-2030 17283135

[B6] Singh SK Hawkins C Clarke ID Squire JA Bayani J Hide T Henkelman RM Cusimano MD Dirks PB Identification of human brain tumour initiating cells Nature 2004 432 7015 396 401 10.1038/nature03128 15549107

[B7] O'Brien CA Pollett A Gallinger S Dick JE A human colon cancer cell capable of initiating tumour growth in immunodeficient mice Nature 2007 445 7123 106 110 10.1038/nature05372 17122772

[B8] Ricci-Vitiani L Lombardi DG Pilozzi E Biffoni M Todaro M Peschle C De Maria R Identification and expansion of human colon-cancer-initiating cells Nature 2007 445 7123 111 115 10.1038/nature05384 17122771

[B9] Miraglia S Godfrey W Yin AH Atkins K Warnke R Holden JT Bray RA Waller EK Buck DW A novel five-transmembrane hematopoietic stem cell antigen: isolation, characterization, and molecular cloning Blood 1997 90 12 5013 5021 9389721

[B10] Mizrak D Brittan M Alison MR CD133: molecule of the moment J Pathol 2008 214 1 3 9 10.1002/path.2283 18067118

[B11] Keysar SB Jimeno A More than markers: biological significance of cancer stem cell-defining molecules Mol Cancer Ther 2010 9 9 2450 2457 10.1158/1535-7163.MCT-10-0530 20716638 PMC3618879

[B12] Lim SC CD24 and human carcinoma: tumor biological aspects Biomed Pharmacother 2005 59 Suppl 2 S351 S354 16507407 10.1016/s0753-3322(05)80076-9

[B13] Sano A Kato H Sakurai S Sakai M Tanaka N Inose T Saito K Sohda M Nakajima M Nakajima T CD24 expression is a novel prognostic factor in esophageal squamous cell carcinoma Ann Surg Oncol 2009 16 2 506 514 10.1245/s10434-008-0252-0 19050962

[B14] Aigner S Sthoeger ZM Fogel M Weber E Zarn J Ruppert M Zeller Y Vestweber D Stahel R Sammar M CD24, a mucin-type glycoprotein, is a ligand for P-selectin on human tumor cells Blood 1997 89 9 3385 3395 9129046

[B15] Baumann P Cremers N Kroese F Orend G Chiquet-Ehrismann R Uede T Yagita H Sleeman JP CD24 expression causes the acquisition of multiple cellular properties associated with tumor growth and metastasis Cancer Res 2005 65 23 10783 10793 10.1158/0008-5472.CAN-05-0619 16322224

[B16] Uekita T Jia L Narisawa-Saito M Yokota J Kiyono T Sakai R CUB domain-containing protein 1 is a novel regulator of anoikis resistance in lung adenocarcinoma Mol Cell Biol 2007 27 21 7649 7660 10.1128/MCB.01246-07 17785447 PMC2169043

[B17] Benes CH Poulogiannis G Cantley LC Soltoff SP The SRC-associated protein CUB Domain-Containing Protein-1 regulates adhesion and motility Oncogene 2012 10.1038/onc.2011.262 PMC3777806 21725358

[B18] Kucia M Reca R Miekus K Wanzeck J Wojakowski W Janowska-Wieczorek A Ratajczak J Ratajczak MZ Trafficking of normal stem cells and metastasis of cancer stem cells involve similar mechanisms: pivotal role of the SDF-1-CXCR4 axis Stem cells (Dayton, Ohio) 2005 23 7 879 894 10.1634/stemcells.2004-0342 15888687

[B19] Workman P Aboagye EO Balkwill F Balmain A Bruder G Chaplin DJ Double JA Everitt J Farningham DA Glennie MJ Guidelines for the welfare and use of animals in cancer research Br J Cancer 2010 102 11 1555 1577 10.1038/sj.bjc.6605642 20502460 PMC2883160

[B20] Fiebig HH Maier A Burger AM Clonogenic assay with established human tumour xenografts: correlation of in vitro to in vivo activity as a basis for anticancer drug discovery Eur J Cancer 2004 40 6 802 820 10.1016/j.ejca.2004.01.009 15120036

[B21] Fiebig HH Schuler J Bausch N Hofmann M Metz T Korrat A Gene signatures developed from patient tumor explants grown in nude mice to predict tumor response to 11 cytotoxic drugs CANCER GENOMICS PROTEOMICS 2007 4 3 197 209 17878523

[B22] Affymetrix Database http://www.affymetrix.com/support/technical/datasheets/human_datasheet.pdf

[B23] Shi L Reid LH Jones WD Shippy R Warrington JA Baker SC Collins PJ de Longueville F Kawasaki ES Lee KY The MicroArray Quality Control (MAQC) project shows inter- and intraplatform reproducibility of gene expression measurements Nat Biotechnol 2006 24 9 1151 1161 10.1038/nbt1239 16964229 PMC3272078

[B24] Zhijin Wu Rafael AI Gentleman Robert Murillo Francisco Martinez Spencer Forrest A Model Based Background Adjustment for Oligonucleotide Expression Arrays 2004 Johns Hopkins University, Department of Biostatistics Working Papers Working Paper 1

[B25] Bengtsson H Ray A Spellman P Speed TP A single-sample method for normalizing and combining full-resolution copy numbers from multiple platforms, labs and analysis methods Bioinformatics (Oxford, England) 2009 25 7 861 867 10.1093/bioinformatics/btp074 PMC2660872 19193730

[B26] Clarke MF Dick JE Dirks PB Eaves CJ Jamieson CH Jones DL Visvader J Weissman IL Wahl GM Cancer stem cells-perspectives on current status and future directions: AACR Workshop on cancer stem cells Cancer Res 2006 66 19 9339 9344 10.1158/0008-5472.CAN-06-3126 16990346

[B27] Reya T Morrison SJ Clarke MF Weissman IL Stem cells, cancer, and cancer stem cells Nature 2001 414 6859 105 111 10.1038/35102167 11689955

[B28] Vermeulen L Sprick MR Kemper K Stassi G Medema JP Cancer stem cells-old concepts, new insights Cell Death Differ 2008 15 6 947 958 10.1038/cdd.2008.20 18259194

[B29] Matsui W Wang Q Barber JP Brennan S Smith BD Borrello I McNiece I Lin L Ambinder RF Peacock C Clonogenic multiple myeloma progenitors, stem cell properties, and drug resistance Cancer Res 2008 68 1 190 197 10.1158/0008-5472.CAN-07-3096 18172311 PMC2603142

[B30] Kelly PN Dakic A Adams JM Nutt SL Strasser A Tumor growth need not be driven by rare cancer stem cells Science (New York, NY) 2007 317 5836 337 10.1126/science.1142596 17641192

[B31] Li L Xie T Stem cell niche: structure and function Annu Rev Cell Dev Biol 2005 21 605 631 10.1146/annurev.cellbio.21.012704.131525 16212509

[B32] Calabrese C Poppleton H Kocak M Hogg TL Fuller C Hamner B Oh EY Gaber MW Finklestein D Allen M A perivascular niche for brain tumor stem cells Cancer Cell 2007 11 1 69 82 10.1016/j.ccr.2006.11.020 17222791

[B33] Vermeulen L De Sousa EMF van der Heijden M Cameron K de Jong JH Borovski T Tuynman JB Todaro M Merz C Rodermond H Wnt activity defines colon cancer stem cells and is regulated by the microenvironment Nat Cell Biol 2003 12 5 468 476 20418870 10.1038/ncb2048

[B34] Todaro M Alea MP Di Stefano AB Cammareri P Vermeulen L Iovino F Tripodo C Russo A Gulotta G Medema JP Colon cancer stem cells dictate tumor growth and resist cell death by production of interleukin-4 Cell Stem Cell 2007 1 4 389 402 10.1016/j.stem.2007.08.001 18371377

[B35] Nikolova T Wu M Brumbarov K Alt R Opitz H Boheler KR Cross M Wobus AM WNT-conditioned media differentially affect the proliferation and differentiation of cord blood-derived CD133+ cells in vitro Differentiation; research in biological diversity 2007 75 2 100 111 10.1111/j.1432-0436.2006.00119.x 17316380

[B36] Reya T Clevers H Wnt signalling in stem cells and cancer Nature 2005 434 7035 843 850 10.1038/nature03319 15829953

[B37] Bruno S Bussolati B Grange C Collino F Graziano ME Ferrando U Camussi G CD133+ renal progenitor cells contribute to tumor angiogenesis Am J Pathol 2006 169 6 2223 2235 10.2353/ajpath.2006.060498 17148683 PMC1762463

[B38] Beier D Hau P Proescholdt M Lohmeier A Wischhusen J Oefner PJ Aigner L Brawanski A Bogdahn U Beier CP CD133(+) and CD133(-) glioblastoma-derived cancer stem cells show differential growth characteristics and molecular profiles Cancer Res 2007 67 9 4010 4015 10.1158/0008-5472.CAN-06-4180 17483311

[B39] Kim J Takeuchi H Lam ST Turner RR Wang HJ Kuo C Foshag L Bilchik AJ Hoon DS Chemokine receptor CXCR4 expression in colorectal cancer patients increases the risk for recurrence and for poor survival J Clin Oncol 2005 23 12 2744 2753 15837989 10.1200/JCO.2005.07.078

[B40] Yang M Reynoso J Bouvet M Hoffman RM A transgenic red fluorescent protein-expressing nude mouse for color-coded imaging of the tumor microenvironment J Cell Biochem 2009 106 2 279 284 10.1002/jcb.21999 19097136 PMC2739131

[B41] Matsusue R Kubo H Hisamori S Okoshi K Takagi H Hida K Nakano K Itami A Kawada K Nagayama S Hepatic stellate cells promote liver metastasis of colon cancer cells by the action of SDF-1/CXCR4 axis Ann Surg Oncol 2009 16 9 2645 2653 10.1245/s10434-009-0599-x 19588204

[B42] Fialkow PJ Singer JW Raskind WH Adamson JW Jacobson RJ Bernstein ID Dow LW Najfeld V Veith R Clonal development, stem-cell differentiation, and clinical remissions in acute nonlymphocytic leukemia N Engl J Med 1987 317 8 468 473 10.1056/NEJM198708203170802 3614291

[B43] Xie W Wang X Du W Liu W Qin X Huang S Detection of molecular targets on the surface of CD34+CD38- bone marrow cells in myelodysplastic syndromes Cytometry A 2010 77 9 840 848 20662087 10.1002/cyto.a.20929

[B44] Scherl-Mostageer M Sommergruber W Abseher R Hauptmann R Ambros P Schweifer N Identification of a novel gene, CDCP1, overexpressed in human colorectal cancer Oncogene 2001 20 32 4402 4408 10.1038/sj.onc.1204566 11466621

[B45] Uekita T Tanaka M Takigahira M Miyazawa Y Nakanishi Y Kanai Y Yanagihara K Sakai R CUB-domain-containing protein 1 regulates peritoneal dissemination of gastric scirrhous carcinoma Am J Pathol 2008 172 6 1729 1739 10.2353/ajpath.2008.070981 18467693 PMC2408431

