# Supplementary figures and images for: Characterization of colon cancer cells: a functional approach characterizing CD133 as a potential stem cell marker
Source: BMC Cancer. 2012 Mar 20;12:96. doi: 10.1186/1471-2407-12-96 (PMC3368744; doi:10.1186/1471-2407-12-96)

percentage of positive cells

■ = CD133  
■ = CD24  
■ = CD44  
■ = CDCP1  
■ = CXCR4

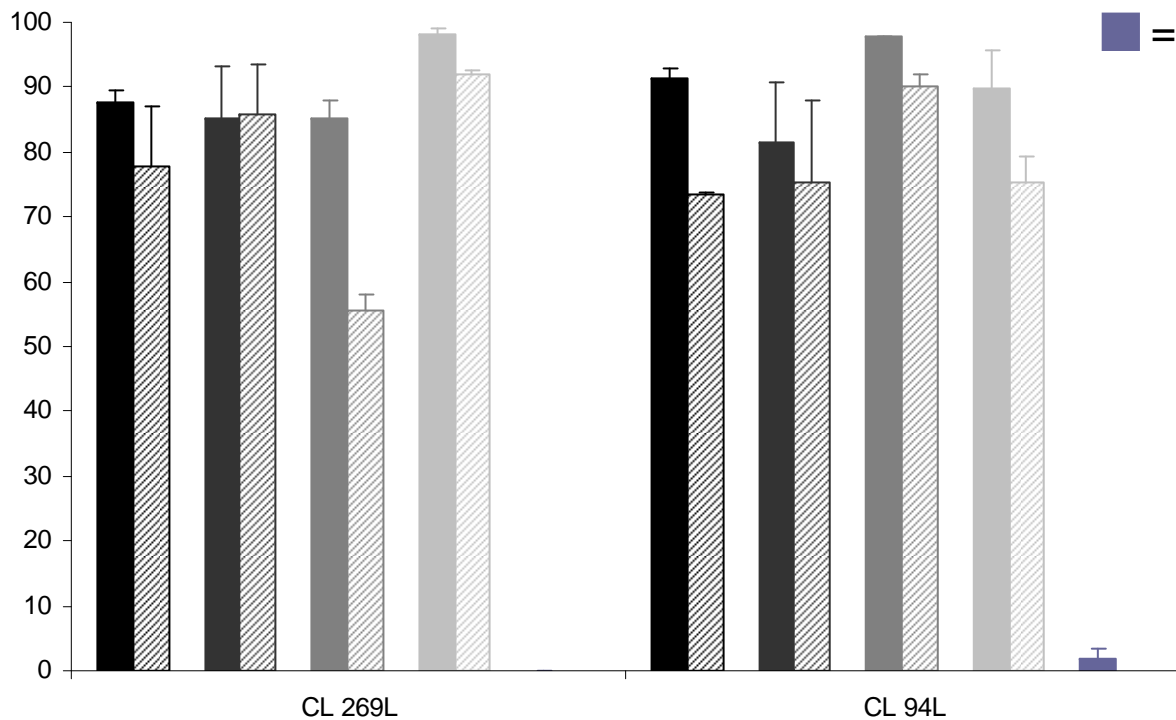

Supplement: Additional file 1 — Figure S7 Expression of five different surface markers on untreated (solid bars) and enzymatically pretreated (shaded bars) colon cancer cell lines 269 L and 94 L. [file 1471-2407-12-96-S1.PDF]
